# Eculizumab improves fatigue in refractory generalized myasthenia gravis

**DOI:** 10.1007/s11136-019-02148-2

**Published:** 2019-03-23

**Authors:** Henning Andersen, Renato Mantegazza, Jing Jing Wang, Fanny O’Brien, Kaushik Patra, James F. Howard, The REGAIN Study Group, Claudio Gabriel Mazia, Miguel Wilken, Fabio Barroso, Juliet Saba, Marcelo Rugiero, Mariela Bettini, Marcelo Chaves, Gonzalo Vidal, Alejandra Dalila Garcia, Jan De Bleecker, Guy Van den Abeele, Kathy de Koning, Katrien De Mey, Rudy Mercelis, Délphine Mahieu, Linda Wagemaekers, Philip Van Damme, Annelies Depreitere, Caroline Schotte, Charlotte Smetcoren, Olivier Stevens, Sien Van Daele, Nicolas Vandenbussche, Annelies Vanhee, Sarah Verjans, Jan Vynckier, Ann D’Hondt, Petra Tilkin, Alzira Alves de Siqueira Carvalho, Igor Dias Brockhausen, David Feder, Daniel Ambrosio, Pamela César, Ana Paula Melo, Renata Martins Ribeiro, Rosana Rocha, Bruno Bezerra Rosa, Thabata Veiga, Luiz Augusto da Silva, Murilo Santos Engel, Jordana Gonçalves Geraldo, Maria da Penha Ananias Morita, Erica Nogueira Coelho, Gabriel Paiva, Marina Pozo, Natalia Prando, Debora Dada Martineli Torres, Cristiani Fernanda Butinhao, Gustavo Duran, Tamires Cristina Gomes da Silva, Luiz Otavio Maia Gonçalves, Lucas Eduardo Pazetto, Tomás Augusto Suriane Fialho, Luciana Renata Cubas Volpe, Luciana Souza Duca, Maurício André Gheller Friedrich, Alexandre Guerreiro, Henrique Mohr, Maurer Pereira Martins, Daiane da Cruz Pacheco, Luciana Ferreira, Ana Paula Macagnan, Graziela Pinto, Aline de Cassia Santos, Acary Souza Bulle Oliveira, Ana Carolina Amaral Andrade, Marcelo Annes, Liene Duarte Silva, Valeria Cavalcante Lino, Wladimir Pinto, Natália Assis, Fernanda Carrara, Carolina Miranda, Iandra Souza, Patricia Fernandes, Zaeem Siddiqi, Cecile Phan, Jeffrey Narayan, Derrick Blackmore, Ashley Mallon, Rikki Roderus, Elizabeth Watt, Stanislav Vohanka, Josef Bednarik, Magda Chmelikova, Marek Cierny, Stanislava Toncrova, Jana Junkerova, Barbora Kurkova, Katarina Reguliova, Olga Zapletalova, Jiri Pitha, Iveta Novakova, Michaela Tyblova, Ivana Jurajdova, Marcela Wolfova, Thomas Harbo, Lotte Vinge, Susanne Krogh, Anita Mogensen, John Vissing, Joan Højgaard, Nanna Witting, Anne Ostergaard Autzen, Jane Pedersen, Juha-Pekka Eralinna, Mikko Laaksonen, Olli Oksaranta, Tuula Harrison, Jaana Eriksson, Csilla Rozsa, Melinda Horvath, Gabor Lovas, Judit Matolcsi, Gyorgyi Szabo, Gedeonne Jakab, Brigitta Szabadosne, Laszlo Vecsei, Livia Dezsi, Edina Varga, Monika Konyane, Giovanni Antonini, Antonella Di Pasquale, Matteo Garibaldi, Stefania Morino, Fernanda Troili, Laura Fionda, Allessandro Filla, Teresa Costabile, Enrico Marano, Francesco Saccà, Angiola Fasanaro, Angela Marsili, Giorgia Puorro, Carlo Antozzi, Silvia Bonanno, Giorgia Camera, Alberta Locatelli, Lorenzo Maggi, Maria Pasanisi, Angela Campanella, Amelia Evoli, Paolo Emilio Alboini, Valentina D’Amato, Raffaele Iorio, Maurizio Inghilleri, Laura Fionda, Vittorio Frasca, Elena Giacomelli, Maria Gori, Diego Lopergolo, Emanuela Onesti, Vittorio Frasca, Maria Gabriele, Akiyuki Uzawa, Tetsuya Kanai, Naoki Kawaguchi, Masahiro Mori, Yoko Kaneko, Akiko Kanzaki, Eri Kobayashi, Hiroyuki Murai, Katsuhisa Masaki, Dai Matsuse, Takuya Matsushita, Taira Uehara, Misa Shimpo, Maki Jingu, Keiko Kikutake, Yumiko Nakamura, Yoshiko Sano, Kimiaki Utsugisawa, Yuriko Nagane, Ikuko Kamegamori, Tomoko Tsuda, Yuko Fujii, Kazumi Futono, Yukiko Ozawa, Aya Mizugami, Yuka Saito, Hidekazu Suzuki, Miyuki Morikawa, Makoto Samukawa, Sachiko Kamakura, Eriko Miyawaki, Hirokazu Shiraishi, Teiichiro Mitazaki, Masakatsu Motomura, Akihiro Mukaino, Shunsuke Yoshimura, Shizuka Asada, Seiko Yoshida, Shoko Amamoto, Tomomi Kobashikawa, Megumi Koga, Yasuko Maeda, Kazumi Takada, Mihoko Takada, Masako Tsurumaru, Yumi Yamashita, Yasushi Suzuki, Tetsuya Akiyama, Koichi Narikawa, Ohito Tano, Kenichi Tsukita, Rikako Kurihara, Fumie Meguro, Yusuke Fukuda, Miwako Sato, Meinoshin Okumura, Soichiro Funaka, Tomohiro Kawamura, Masayuki Makamori, Masanori Takahashi, Namie Taichi, Tomoya Hasuike, Eriko Higuchi, Hisako Kobayashi, Kaori Osakada, Tomihiro Imai, Emiko Tsuda, Shun Shimohama, Takashi Hayashi, Shin Hisahara, Jun Kawamata, Takashi Murahara, Masaki Saitoh, Shuichiro Suzuki, Daisuke Yamamoto, Yoko Ishiyama, Naoko Ishiyama, Mayuko Noshiro, Rumi Takeyama, Kaori Uwasa, Ikuko Yasuda, Anneke van der Kooi, Marianne de Visser, Tamar Gibson, Byung-Jo Kim, Chang Nyoung Lee, Yong Seo Koo, Hung Youl Seok, Hoo Nam Kang, HyeJin Ra, Byoung Joon Kim, Eun Bin Cho, MiSong Choi, HyeLim Lee, Ju-Hong Min, Jinmyoung Seok, JiEun Lee, Da Yoon Koh, JuYoung Kwon, SangAe Park, Eun Hwa Choi, Yoon-Ho Hong, So-Hyun Ahn, Dae Lim Koo, Jae-Sung Lim, ChaeWon Shin, Ji Ye Hwang, Miri Kim, Seung Min Kim, Ha-Neul Jeong, JinWoo Jung, Yool-hee Kim, Hyung Seok Lee, Ha Young Shin, Eun Bi Hwang, Miju Shin, Carlos Casasnovas, Maria Antonia Alberti Aguilo, Christian Homedes-Pedret, Natalia Julia Palacios, Laura Diez Porras, Valentina Velez Santamaria, Ana Lazaro, Exuperio Diez Tejedor, Pilar Gomez Salcedo, Mireya Fernandez-Fournier, Pedro Lopez Ruiz, Francisco Javier Rodriguez de Rivera, Maria Sastre, Josep Gamez, Pilar Sune, Maria Salvado, Gisela Gili, Gonzalo Mazuela, Isabel Illa, Elena Cortes Vicente, Jordi Diaz-Manera, Luis Antonio Querol Gutierrez, Ricardo Rojas Garcia, Nuria Vidal, Elisabet Arribas-Ibar, Fredrik Piehl, Albert Hietala, Lena Bjarbo, Ihsan Sengun, Arzu Meherremova, Pinar Ozcelik, Bengu Balkan, Celal Tuga, Muzeyyen Ugur, Sevim Erdem-Ozdamar, Can Ebru Bekircan-Kurt, Nazire Pinar Acar, Ezgi Yilmaz, Yagmur Caliskan, Gulsah Orsel, Husnu Efendi, Seda Aydinlik, Hakan Cavus, Ayse Kutlu, Gulsar Becerikli, Cansu Semiz, Ozlem Tun, Murat Terzi, Baki Dogan, Musa Kazim Onar, Sedat Sen, Tugce Kirbas Cavdar, Adife Veske, Fiona Norwood, Aikaterini Dimitriou, Jakit Gollogly, Mohamed Mahdi-Rogers, Arshira Seddigh, Giannis Sokratous, Gal Maier, Faisal Sohail, Saiju Jacob, Girija Sadalage, Pravin Torane, Claire Brown, Amna Shah, Sivakumar Sathasivam, Heike Arndt, Debbie Davies, Dave Watling, Anthony Amato, Thomas Cochrane, Mohammed Salajegheh, Kristen Roe, Katherine Amato, Shirli Toska, Gil Wolfe, Nicholas Silvestri, Kara Patrick, Karen Zakalik, Jonathan Katz, Robert Miller, Marguerite Engel, Dallas Forshew, Elena Bravver, Benjamin Brooks, Sarah Plevka, Maryanne Burdette, Scott Cunningham, Mohammad Sanjak, Megan Kramer, Joanne Nemeth, Clara Schommer, Scott Tierney, Vern Juel, Jeffrey Guptill, Lisa Hobson-Webb, Janice Massey, Kate Beck, Donna Carnes, John Loor, Amanda Anderson, Robert Pascuzzi, Cynthia Bodkin, John Kincaid, Riley Snook, Sandra Guingrich, Angela Micheels, Vinay Chaudhry, Andrea Corse, Betsy Mosmiller, Andrea Kelley, Doreen Ho, Jayashri Srinivasan, Michal Vytopil, Jordan Jara, Nicholas Ventura, Stephanie Scala, Cynthia Carter, Craig Donahue, Carol Herbert, Elaine Weiner, Sharmeen Alam, Jonathan McKinnon, Laura Haar, Naya McKinnon, Karan Alcon, Kaitlyn McKenna, Nadia Sattar, Kevin Daniels, Dennis Jeffery, John Kissel, Miriam Freimer, Joseph Chad Hoyle, Julie Agriesti, Sharon Chelnick, Louisa Mezache, Colleen Pineda, Filiz Muharrem, Chafic Karam, Julie Khoury, Tessa Marburger, Harpreet Kaur, Diana Dimitrova, James Gilchrist, Brajesh Agrawal, Mona Elsayed, Stephanie Kohlrus, Angela Andoin, Taylor Darnell, Laura Golden, Barbara Lokaitis, Jenna Seelback, Srikanth Muppidi, Neelam Goyal, Sarada Sakamuri, Yuen T. So, Shirley Paulose, Sabrina Pol, Lesly Welsh, Ratna Bhavaraju-Sanka, Alejandro Tobon Gonzales, Lorraine Dishman, Floyd Jones, Anna Gonzalez, Patricia Padilla, Amy Saklad, Marcela Silva, Sharon Nations, Jaya Trivedi, Steve Hopkins, Mohamed Kazamel, Mohammad Alsharabati, Liang Lu, Kenkichi Nozaki, Sandi Mumfrey-Thomas, Amy Woodall, Tahseen Mozaffar, Tiyonnoh Cash, Namita Goyal, Gulmohor Roy, Veena Mathew, Fatima Maqsood, Brian Minton, H. James Jones, Jeffrey Rosenfeld, Rebekah Garcia, Laura Echevarria, Sonia Garcia, Michael Pulley, Shachie Aranke, Alan Ross Berger, Jaimin Shah, Yasmeen Shabbir, Lisa Smith, Mary Varghese, Laurie Gutmann, Ludwig Gutmann, Nivedita Jerath, Christopher Nance, Andrea Swenson, Heena Olalde, Nicole Kressin, Jeri Sieren, Richard Barohn, Mazen Dimachkie, Melanie Glenn, April McVey, Mamatha Pasnoor, Jeffery Statland, Junxia Wang, Tina Liu, Kelley Emmons, Nicole Jenci, Jerry Locheke, Alex Fondaw, Kathryn Johns, Gabrielle Rico, Maureen Walsh, Laura Herbelin, Charlene Hafer-Macko, Justin Kwan, Lindsay Zilliox, Karen Callison, Valerie Young, Beth DiSanzo, Kerry Naunton, Michael Benatar, Martin Bilsker, Khema Sharma, Anne Cooley, Eliana Reyes, Sara-Claude Michon, Danielle Sheldon, Julie Steele, Chafic Karam, Manisha Chopra, Rebecca Traub, Tuan Vu, Lara Katzin, Terry McClain, Brittany Harvey, Adam Hart, Kristin Huynh, Said Beydoun, Amaiak Chilingaryan, Victor Doan, Brian Droker, Hui Gong, Sanaz Karimi, Frank Lin, Terry McClain, Krishna Pokala, Akshay Shah, Anh Tran, Salma Akhter, Ali Malekniazi, Rup Tandan, Michael Hehir, Waqar Waheed, Shannon Lucy, Michael Weiss, Jane Distad, Susan Strom, Sharon Downing, Bryan Kim, Tulio Bertorini, Thomas Arnold, Kendrick Hendersen, Rekha Pillai, Ye Liu, Lauren Wheeler, Jasmine Hewlett, Mollie Vanderhook, Richard Nowak, Daniel Dicapua, Benison Keung, Aditya Kumar, Huned Patwa, Kimberly Robeson, Irene Yang, Joan Nye, Hong Vu

**Affiliations:** 1 Department of Neurology, Aarhus University Hospital, Indgang J, Plan 5, Krydspunkt 504, Palle Juul-Jensens Boulevard, 8200 Aarhus N, Denmark; 2 Foundation of the Carlo Besta Neurological Institute, IRCCS, Milan, Italy; 3 Formerly of Alexion Pharmaceuticals, Inc., New Haven, CT USA; 4 Alexion Pharmaceuticals, Inc., New Haven, CT USA; 5 Department of Neurology, UNC School of Medicine, University of North Carolina at Chapel Hill, Chapel Hill, NC USA

**Keywords:** Eculizumab, Myasthenia gravis, Fatigue, Neuro-QOL Fatigue, Quality of life, Terminal complement inhibition, Complement

## Abstract

**Purpose:**

To evaluate the effect of eculizumab on perceived fatigue in patients with anti-acetylcholine receptor antibody-positive, refractory, generalized myasthenia gravis (MG) using the Quality of Life in Neurological Disorders (Neuro-QOL) Fatigue subscale, and to evaluate correlations between improvements in Neuro-QOL Fatigue and other clinical endpoints.

**Methods:**

Neuro-QOL Fatigue, MG Activities of Daily Living (MG-ADL), Quantitative MG (QMG), and the 15-item MG Quality of Life (MG-QOL15) scales were administered during the phase 3, randomized, placebo-controlled REGAIN study (eculizumab, *n* = 62; placebo, *n* = 63) and subsequent open-label extension (OLE). Data were analyzed using repeated-measures models. Correlations between changes in Neuro-QOL Fatigue and in MG-ADL, QMG, and MG-QOL15 scores were determined at REGAIN week 26.

**Results:**

At REGAIN week 26, eculizumab-treated patients showed significantly greater improvements in Neuro-QOL Fatigue scores than placebo-treated patients (consistent with improvements in MG-ADL, QMG, and MG-QOL15 scores previously reported in REGAIN). Improvements with eculizumab were sustained through OLE week 52. Correlations between Neuro-QOL Fatigue and MG-QOL15, MG-ADL, and QMG scores were strong for eculizumab-treated patients at REGAIN week 26, and strong, moderate, and weak, respectively, for placebo-treated patients.

**Conclusions:**

Compared with placebo, eculizumab was associated with improvements in perceived fatigue that strongly correlated with improvements in MG-specific outcome measures.

**Trial ID Registration**: NCT01997229, NCT02301624.

**Electronic supplementary material:**

The online version of this article (10.1007/s11136-019-02148-2) contains supplementary material, which is available to authorized users.

## Introduction

Patients with anti-acetylcholine receptor antibody-positive (AChR+), refractory, generalized myasthenia gravis (gMG) experience muscle weakness, which is associated with complement-mediated damage at the neuromuscular junction [1, 2]. Patients with MG experience muscle fatigability (difficulty initiating or sustaining muscle activities) [1, 3]. Perceived fatigue (a feeling of tiredness, lack of energy, and difficulty concentrating; referred to as fatigue hereafter) is also an important clinical issue for these patients and may be distinct from muscle weakness and fatigability [3–7].

Validated MG-specific assessments used to measure disease severity and response to intervention include the patient-reported MG Activities of Daily Living (MG-ADL) and 15-item MG Quality of Life (MG-QOL15) scales, and the physician-completed Quantitative MG (QMG) assessment of muscle strength [8–10]. None of these evaluate the impact of fatigue in gMG. The Quality of Life in Neurological Disorders (Neuro-QOL) assessment is designed to measure health-related quality of life across 13 subscales in patients with neurological conditions [11]. Neuro-QOL has been validated in a range of disorders, including multiple sclerosis, Parkinson’s disease, and epilepsy, but not in MG [12–14]. The Neuro-QOL Fatigue subscale comprises a 19-item, patient self-assessment of fatigue that encompasses ‘sensations ranging from tiredness to an overwhelming, debilitating, and sustained sense of exhaustion that decrease one’s capacity for physical, functional, social, and mental activities’ [15]. A short form Neuro-QOL Fatigue subscale is also available; this was recently validated in patients with MG [7].

Eculizumab is a humanized monoclonal antibody that binds to complement protein C5, inhibiting the activation of the terminal complement component proteins that are believed to mediate damage at the neuromuscular junction in AChR+ gMG [2, 16]. In the phase 3 REGAIN study, eculizumab demonstrated clinically meaningful improvements in patients with AChR+ refractory gMG, evaluated using MG-ADL, QMG, and MG-QOL15 assessments (NCT01997229) [17].

The current analysis investigated the effect of eculizumab versus placebo on fatigue in patients with AChR+ refractory gMG, and assessed the relationship between fatigue and MG-specific measures. We used the Neuro-QOL Fatigue subscale to evaluate changes in patients’ self-reported assessment of fatigue during REGAIN and its open-label extension study (NCT02301624; interim analysis, 31 December 2017) [18]. We also measured the correlation between outcomes measured using the Neuro-QOL Fatigue subscale and the MG-ADL, QMG, and MG-QOL15 scales in REGAIN.

## Methods

### Study design

REGAIN was a 26-week, phase 3, randomized, double-blind, placebo-controlled study evaluating eculizumab efficacy and safety [17]. Briefly, patients were randomized 1:1 to receive eculizumab (*n* = 62; induction: 900 mg for 4 weeks; maintenance: 1200 mg at week 4 and then every 2 weeks until week 26) or placebo (*n* = 63) [17]. The primary endpoint was the change in MG-ADL total score with eculizumab versus placebo.

The Neuro-QOL Fatigue subscale was used to assess fatigue, and the MG-ADL, QMG, and MG-QOL15 scales were used to measure MG-specific activities of daily living, muscle strength, and quality of life, respectively. In REGAIN, all assessments were performed at baseline (day 1), every 4 weeks to week 20, and at week 26. MG-ADL and QMG assessments were also conducted at weeks 1, 2, and 3. Details of scoring for MG-ADL, QMG, and MG-QOL15 during REGAIN have been reported previously; briefly, for each measure, a reduction from baseline total score indicates improvement [17]. The response scale for Neuro-QOL Fatigue items was: 1 = never; 2 = rarely; 3 = sometimes; 4 = often; and 5 = always. The range of potential total scores was 19–95. Higher Neuro-QOL Fatigue subscale total scores indicate greater fatigue; a reduction from baseline total score indicates improvement [15].

Patients who completed REGAIN could enroll into the extension study [18]. Following a 4-week blinded induction phase, all patients received open-label eculizumab (1200 mg) at week 4 and every 2 weeks thereafter. Assessments occurred as in REGAIN to week 26, then at week 40, week 52, and every 6 months [18].

### Statistical analysis

**Fig. 1:**
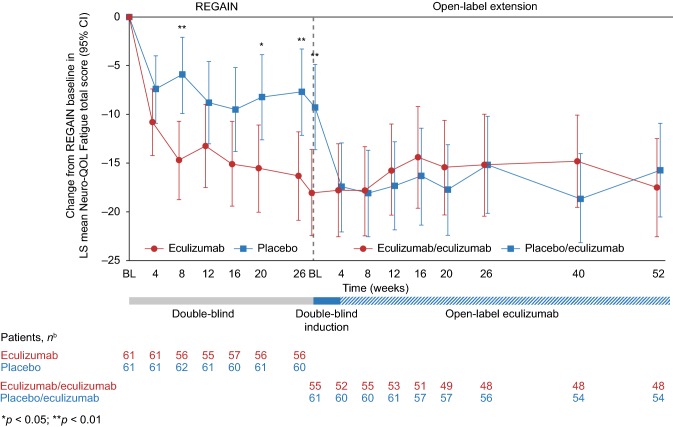
Change in Neuro-QOL Fatigue subscale total score from REGAIN baseline to week 52 of the open-label study using a repeated-measures model.^a^^a^A repeated-measures model using the restricted maximum likelihood for the changes from baseline was used to compare the two treatment groups at each assessment visit and over time. The model included the following terms: treatment, visit, treatment by visit interaction, pooled Myasthenia Gravis Foundation of America (MGFA) randomization stratification variable (based on their MGFA classification at screening, patients were assigned to one of two categories: IIa/IIIa/IVa [a, symptoms predominantly affecting limb or axial muscles, or both] or IIb/IIIb/IVb [b, symptoms predominantly affecting oropharyngeal or respiratory muscles, or both]), and Neuro-QOL Fatigue total score at baseline. ^b^Number of patients who completed the assessment at each time point. Some patients did not complete all items of the questionnaire at every timepoint. When this occurred, total scores could not be computed at that time point. Missing scores were not imputed. *BL* baseline, *CI* confidence interval, *Neuro-QOL Fatigue* Quality of Life in Neurological Disorders Fatigue subscale, *REGAIN* Eculizumab for REfractory GenerAlIzed MyastheNia Gravis

A repeated-measures model was used to test whether changes in Neuro-QOL Fatigue total scores from baseline to REGAIN week 26 for eculizumab and placebo were equal (Fig. 1). Model-estimated changes from REGAIN baseline data were reported up to open-label study week 52 (interim analysis, 31 December 2017) (Fig. 1). To evaluate the treatment effect on Neuro-QOL Fatigue subscale individual items at REGAIN week 26, a repeated-measures proportional odds model was implemented using the GEEORD SAS Macro (Table 2) [19]. Missing scores were not imputed in either model.

**Fig. 2:**
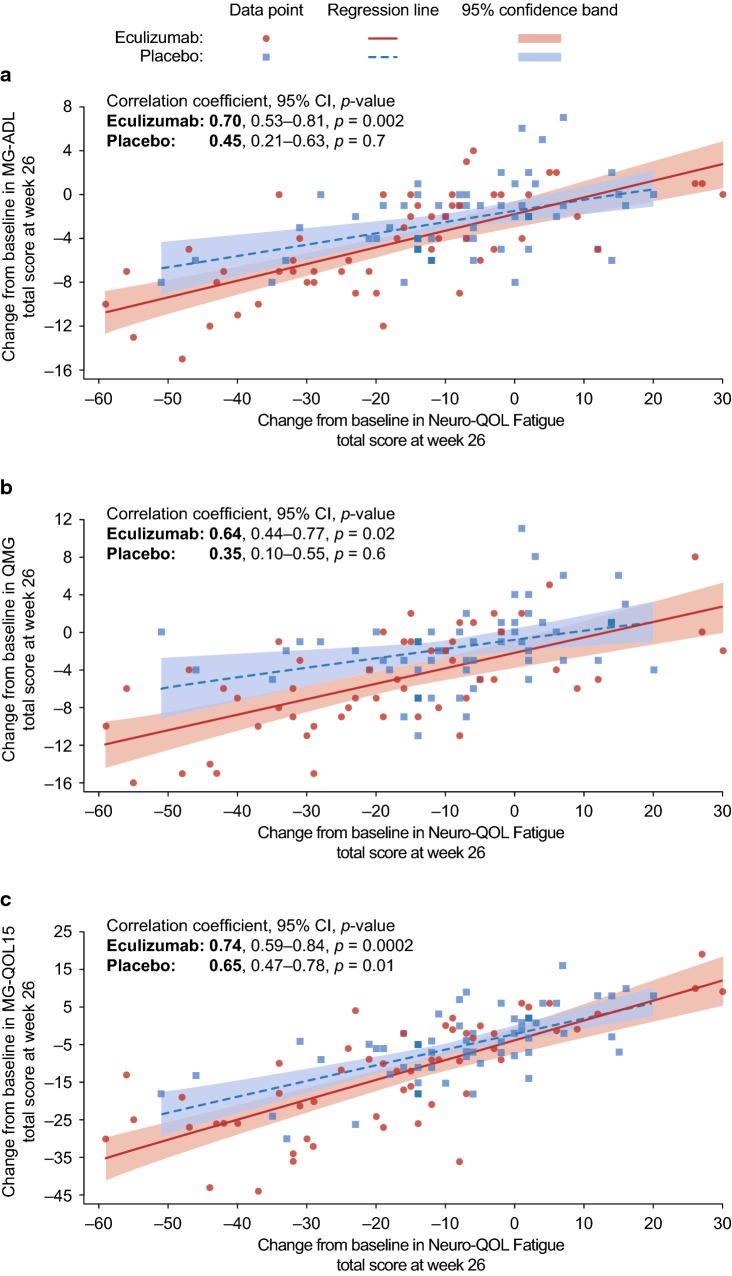
Correlation between change from REGAIN baseline to week 26, in Neuro-QOL Fatigue subscale total score and **a** MG-ADL, **b** QMG, and **c** MG-QOL15 total scores by treatment group (eculizumab, *n* = 54; placebo, *n* = 58). Improvements in Neuro-QOL Fatigue total score correlated with improvements in **a** MG-ADL, **b** QMG, and **c** MG-QOL15 total scores more strongly in patients treated with eculizumab than in those who received placebo. Within treatment arms, the null hypothesis was *r* = 0.4 (the lower limit for moderate correlations). *CI* confidence interval, *MG-ADL* Myasthenia Gravis-Activities of Daily Living, *MG-QOL15* 15-item Myasthenia Gravis Quality of Life, *Neuro-QOL Fatigue* Quality of Life in Neurological Disorders Fatigue subscale, *QMG* quantitative myasthenia gravis

Pearson correlation coefficients (*r*) were calculated for the treatment arms using observed changes from baseline to REGAIN week 26 for Neuro-QOL Fatigue total scores versus observed changes for MG-ADL, QMG, and MG-QOL15 total scores (Fig. 2). The strength of association for absolute values of *r* was classified as follows: very weak, 0–0.19; weak, 0.2–0.39; moderate, 0.40–0.59; strong, 0.6–0.79; and very strong, 0.8–1 [20]. Within each treatment arm, the null hypothesis was *r* = 0.4 (the lower limit for moderate correlations). For all evaluations, statistical significance was established using a two-sided *p* value of less than 0.05, without any multiplicity adjustment.

## Results

Baseline demographics and characteristics were similar between treatment groups, with the exception of a higher proportion of Asian patients in the placebo group (Table 1). All patients were assessed using the Neuro-QOL Fatigue subscale (Table 1); mean (standard deviation) baseline total score was 64.1 (14.1) for the eculizumab group and 61.8 (15.9) for the placebo group.

**Table 1:** Baseline demographics and characteristics of patients in REGAIN

	Eculizumab (*n* = 62)	Placebo (*n* = 63)
Age at diagnosis, years, mean (SD)	38.0 (17.8)	38.1 (19.6)
Age at first study dose, years, mean (SD)	47.5 (15.7)	46.9 (18.0)
Sex, *n* (%)		
Male	21 (34%)	22 (35%)
Female	41 (66%)	41 (65%)
Race, *n* (%)		
Asian	3 (5%)	16 (25%)
Black or African American	0	3 (5%)
White	53 (85%)	42 (67%)
Other	6 (10%)	2 (3%)
BMI, kg/m^2^, mean (SD)	31.4 (9.0)	30.5 (8.4)
Myasthenia gravis duration, years, mean (SD)	9.9 (8.1)	9.2 (8.4)
Neuro-QOL Fatigue total score, mean (SD); median^a^	64.1 (14.1); 64.0	61.8 (15.9); 64.0
Neuro-QOL Fatigue item score, mean (SD); median^a^		
I felt exhausted	3.6 (0.82); 4.0	3.5 (1.09); 4.0
I felt that I had no energy	3.7 (1.04); 4.0	3.7 (0.98); 4.0
I felt fatigued	3.8 (0.88); 4.0	3.7 (0.91); 4.0
I was too tired to do my household chores	3.5 (1.01); 4.0	3.3 (1.03); 3.0
I was too tired to leave the house	3.2 (1.01); 3.0	3.2 (0.99); 3.0
I was frustrated by being too tired to do the things I wanted to do	3.7 (1.08); 4.0	3.4 (1.21); 3.0
I felt tired	3.8 (0.80); 4.0	3.8 (0.97); 4.0
I had to limit my social activity because I was tired	3.6 (1.00); 4.0	3.4 (1.13); 4.0
I needed help doing my usual activities because of my fatigue	3.0 (1.24); 3.0	2.9 (1.22); 3.0
I needed to sleep during the day	3.3 (1.29); 3.0	3.4 (1.23); 4.0
I had trouble starting things because I was too tired	3.3 (0.89); 3.0	3.1 (1.13); 3.0
I had trouble finishing things because I was too tired	3.5 (0.99); 4.0	3.2 (1.06); 3.0
I was too tired to take a short walk	3.4 (1.19); 4.0	3.1 (1.29); 3.0
I was too tired to eat	2.6 (1.07); 3.0	2.3 (1.09); 2.0
I was so tired that I had to rest during the day	3.7 (1.06); 4.0	3.6 (1.07); 4.0
I felt weak all over	3.3 (1.07); 3.0	3.3 (1.13); 3.0
I needed help doing my usual activities because of weakness	2.9 (1.24); 3.0	2.9 (1.18); 3.0
I had to limit my social activity because I was physically weak	3.3 (1.23); 3.0	3.3 (1.04); 3.0
I had to force myself to get up and do things because I was physically too weak	3.0 (1.18); 3.0	2.9 (1.17); 3.0

*SD* standard deviation

^a^Some patients did not complete all items of the questionnaire. When this occurred, total scores could not be computed and the specific item score is missing for those patients

Eculizumab was associated with greater improvements in fatigue than placebo. At REGAIN week 26, patients receiving eculizumab had a model-estimated mean change from baseline Neuro-QOL Fatigue total score of − 16.3 (95% confidence interval [CI]: − 20.8, − 11.8) versus − 7.7 (95% CI − 12.1, − 3.3) for placebo (model-estimated mean difference − 8.6 [95% CI − 14.8, − 2.3; *p* = 0.0081]) (Fig. 1). By week 4 of the open-label study, patients who had received eculizumab in both studies (eculizumab/eculizumab arm) had reductions from REGAIN baseline of − 17.8 (95% CI − 22.5, − 13.0) compared with − 17.4 (95% CI − 22.0, − 12.9) for patients who received placebo during REGAIN and eculizumab during the open-label study (placebo/eculizumab arm) (Fig. 1). Improvements in Neuro-QOL Fatigue score, from REGAIN baseline, were maintained through open-label study week 52 (–17.5 [95% CI − 22.5, − 12.5] and − 15.7 [95% CI − 20.5, − 10.9] for the eculizumab/eculizumab and placebo/eculizumab arms, respectively).

At REGAIN week 26, the proportional odds ratios for all 19 Neuro-QOL Fatigue items were greater than 1, indicating that a greater proportion of eculizumab-treated patients than placebo-treated patients selected lower scoring responses (i.e., more selected ‘never’ or ‘rarely,’ and fewer selected ‘often’ or ‘always’) (Table 2). This was statistically significant for 15 of 19 items (range of common odds ratios with *p* < 0.05: 1.8–2.9; Table 2), indicating that eculizumab treatment was associated with milder fatigue than placebo.

**Table 2:** Neuro-QOL Fatigue individual item responses and results from a repeated-measures proportional odds model at REGAIN week 26

Parameter	Treatment	Observed responses for Neuro-QOL Fatigue items at REGAIN week 26					Repeated-measures model estimate for REGAIN week 26		
		1 = Never, *n* (%)	2 = Rarely, *n* (%)	3 = Sometimes, *n* (%)	4 = Often, *n* (%)	5 = Always, *n* (%)	Proportional odds ratio (95% CI)^b^	*p* value for odds ratio^b,c^	*p* value for proportional odds assumption^d^
I felt exhausted	ECU (*n*^*a*^ = 57)	9 (15.8)	14 (24.6)	19 (33.3)	13 (22.8)	2 (3.5)	2.4 (1.3, 4.4)	0.0038	0.1099
	PLC (*n*^*a*^ = 60)	5 (8.3)	9 (15.0)	25 (41.7)	16 (26.7)	5 (8.3)			
I felt that I had no energy	ECU (*n*^a^ = 57)	9 (15.8)	15 (26.3)	21 (36.8)	9 (15.8)	3 (5.3)	1.9 (1.1, 3.5)	0.0322	0.5692
	PLC (*n*^a^ = 60)	5 (8.3)	10 (16.7)	23 (38.3)	15 (25.0)	7 (11.7)			
I felt fatigued	ECU (*n*^a^ = 57)	7 (12.3)	16 (28.1)	22 (38.6)	8 (14.0)	4 (7.0)	1.8 (1.0, 3.4)	0.0467	0.3357
	PLC (*n*^a^ = 60)	2 (3.3)	14 (23.3)	24 (40.0)	15 (25.0)	5 (8.3)			
I was too tired to do my household chores	ECU (*n*^a^ = 56)	16 (28.6)	15 (26.8)	13 (23.2)	7 (12.5)	5 (8.9)	1.9 (1.0, 3.4)	0.0437	0.6454
	PLC (*n*^a^ = 60)	11 (18.3)	11 (18.3)	19 (31.7)	17 (28.3)	2 (3.3)			
I was too tired to leave the house	ECU (*n*^a^ = 57)	22 (38.6)	12 (21.1)	12 (21.1)	8 (14.0)	3 (5.3)	2.5 (1.4, 4.7)	0.0033	0.2162
	PLC (*n*^a^ = 60)	12 (20.0)	11 (18.3)	20 (33.3)	15 (25.0)	2 (3.3)			
I was frustrated by being too tired to do the things I wanted to do	ECU (*n*^a^ = 57)	20 (35.1)	14 (24.6)	11 (19.3)	8 (14.0)	4 (7.0)	2.5 (1.4, 4.5)	0.0032	0.4059
	PLC (*n*^a^ = 60)	14 (23.3)	7 (11.7)	16 (26.7)	18 (30.0)	5 (8.3)			
I felt tired	ECU (*n*^a^ = 57)	9 (15.8)	9 (15.8)	22 (38.6)	12 (21.1)	5 (8.8)	2.3 (1.2, 4.4)	0.0092	0.3737
	PLC (*n*^a^ = 60)	0 (0.0)	9 (15.0)	26 (43.3)	17 (28.3)	8 (13.3)			
I had to limit my social activity because I was tired	ECU (*n*^a^ = 57)	15 (26.3)	16 (28.1)	13 (22.8)	9 (15.8)	4 (7.0)	2.6 (1.4, 4.7)	0.0026	0.6225
	PLC (*n*^a^ = 60)	8 (13.3)	11 (18.3)	18 (30.0)	17 (28.3)	6 (10.0)			
I needed help doing my usual activities because of my fatigue	ECU (*n*^a^ = 57)	26 (45.6)	10 (17.5)	10 (17.5)	7 (12.3)	4 (7.0)	2.3 (1.2, 4.2)	0.0104	0.5279
	PLC (*n*^a^ = 60)	14 (23.3)	11 (18.3)	20 (33.3)	12 (20.0)	3 (5.0)			
I needed to sleep during the day	ECU (*n*^a^ = 57)	14 (24.6)	14 (24.6)	13 (22.8)	10 (17.5)	6 (10.5)	1.5 (0.8, 2.7)	0.2164	0.4892
	PLC (*n*^a^ = 60)	12 (20.0)	7 (11.7)	16 (26.7)	15 (25.0)	10 (16.7)			
I had trouble starting things because I was too tired	ECU (*n*^a^ = 57)	16 (28.1)	18 (31.6)	13 (22.8)	6 (10.5)	4 (7.0)	2.2 (1.2, 4.1)	0.0096	0.1930
	PLC (*n*^a^ = 60)	15 (25.0)	7 (11.7)	18 (30.0)	13 (21.7)	7 (11.7)			
I had trouble finishing things because I was too tired	ECU (*n*^a^ = 57)	20 (35.1)	13 (22.8)	13 (22.8)	8 (14.0)	3 (5.3)	2.5 (1.4, 4.5)	0.0027	0.8333
	PLC (*n*^a^ = 60)	12 (20.0)	11 (18.3)	18 (30.0)	13 (21.7)	6 (10.0)			
I was too tired to take a short walk	ECU (*n*^a^ = 57)	19 (33.3)	14 (24.6)	11 (19.3)	9 (15.8)	4 (7.0)	1.5 (0.8, 2.7)	0.1896	0.9608
	PLC (*n*^a^ = 60)	17 (28.3)	10 (16.7)	15 (25.0)	14 (23.3)	4 (6.7)			
I was too tired to eat	ECU (*n*^a^ = 57)	30 (52.6)	12 (21.1)	10 (17.5)	1 (1.8)	4 (7.0)	1.2 (0.6, 2.3)	0.5365	0.9093
	PLC (*n*^a^ = 60)	25 (41.7)	17 (28.3)	11 (18.3)	7 (11.7)	0 (0.0)			
I was so tired that I had to rest during the day	ECU (*n*^a^ = 57)	10 (17.5)	11 (19.3)	13 (22.8)	17 (29.8)	6 (10.5)	1.2 (0.7, 2.3)	0.4697	0.4873
	PLC (*n*^a^ = 60)	9 (15.0)	8 (13.3)	19 (31.7)	14 (23.3)	10 (16.7)			
I felt weak all over	ECU (*n*^a^ = 57)	23 (40.4)	9 (15.8)	13 (22.8)	7 (12.3)	5 (8.8)	2.5 (1.4, 4.7)	0.0032	0.4309
	PLC (*n*^a^ = 60)	12 (20.0)	12 (20.0)	14 (23.3)	17 (28.3)	5 (8.3)			
I needed help doing my usual activities because of weakness	ECU (*n*^a^ = 57)	26 (45.6)	12 (21.1)	10 (17.5)	6 (10.5)	3 (5.3)	2.3 (1.2, 4.2)	0.0095	0.3340
	PLC (*n*^a^ = 60)	14 (23.3)	13 (21.7)	17 (28.3)	14 (23.3)	2 (3.3)			
I had to limit my social activity because I was physically weak	ECU (*n*^a^ = 57)	19 (33.3)	18 (31.6)	6 (10.5)	10 (17.5)	4 (7.0)	2.9 (1.6, 5.3)	0.0006	0.1119
	PLC (*n*^a^ = 60)	10 (16.7)	8 (13.3)	20 (33.3)	15 (25.0)	7 (11.7)			
I had to force myself to get up and do things because I was physically too weak	ECU (*n*^a^ = 57)	25 (43.9)	15 (26.3)	9 (15.8)	5 (8.8)	3 (5.3)	2.1 (1.1, 4.0)	0.0172	0.0927
	PLC (*n*^a^ = 60)	19 (31.7)	7 (11.7)	18 (30.0)	15 (25.0)	1 (1.7)			

The *p* values associated with the proportional odds assumption indicated that this assumption was upheld for each item

*CI* confidence interval, *ECU* eculizumab, *Neuro-QOL* Quality of Life in Neurological Disorders, *PLC* placebo

^a^Number of patients who completed the assessment at week 26; missing scores were not imputed for patients who did not complete the assessment (ECU, *n* = 5 [*n* = 6 for ‘I was too tired to do my household chores’]; PLC, *n* = 3)

^b^Proportional odds ratios and *p *values are based on a repeated-measures ordinal regression (implemented using the GEEORD SAS Macro). The model has the ordinal response (for each parameter) at each visit as the dependent variable with terms for treatment, visit, and treatment by visit interaction, assuming exchangeable correlation structure. A higher odds ratio indicates that eculizumab-treated patients are more likely to answer in the lower response categories than those receiving placebo at week 26 for that item

^c^*p* values are not adjusted for multiple comparisons

^d^A *p *value for proportional odds assumption < 0.05 may indicate non-proportionality

For the eculizumab group, there were significant correlations (i.e., different from *r* = 0.4) for changes in total score from baseline to REGAIN week 26 between Neuro-QOL Fatigue and MG-QOL15 (*r* = 0.74), MG-ADL (*r* = 0.70), and QMG (*r* = 0.64) (Fig. 2). For the placebo-treated group, a strong correlation for change from baseline to REGAIN week 26 was observed between Neuro-QOL Fatigue and MG-QOL15 (*r* = 0.65); conversely, correlations between Neuro-QOL Fatigue and QMG and MG-ADL were not significantly different from *r* = 0.4 (*r* = 0.45 [moderate] and *r* = 0.35 [weak]) (Fig. 2).

## Discussion

This was the first use of the extended Neuro-QOL Fatigue subscale in patients with AChR+ refractory gMG. In REGAIN, levels of fatigue at baseline were generally consistent with previously published reports for MG; however, in these previous studies, fatigue was assessed using a variety of measures, and patients were not explicitly refractory to treatment [6, 7, 21].

At REGAIN week 26, eculizumab-treated patients were approximately twice as likely to report improved scores for the 15 of 19 subscale items where significant differences were observed, compared with placebo (range for odds ratios: 1.8–2.9; Table 2).

Patients who received eculizumab for the first time during the open-label study had similar improvements in fatigue to those seen with eculizumab during REGAIN. These were sustained through to week 52 of the open-label study and were consistent with previously reported improvements in MG-validated measures of disease burden (QMG, MG-ADL, and MG-QOL15) [17, 22]. The consistently strong, positive correlations between changes in Neuro-QOL Fatigue and MG-QOL15, MG-ADL, and QMG observed in eculizumab-treated patients suggest that the effect of eculizumab on fatigue is a valid clinical outcome in this patient population. The greater variability in observed correlations in placebo-treated patients may reflect the degree of similarity between the items assessed by the Neuro-QOL Fatigue subscale and those assessed by the MG-QOL15 (most similar, strong correlation), the MG-ADL (some similarities, moderate correlation), and the QMG (least similar, weak correlation).

Our findings suggest that the Neuro-QOL Fatigue subscale may be used to assess the clinical impact of perceived fatigue and the effect of therapeutic interventions in patients with AChR+ refractory gMG. Use of this scale demonstrated that eculizumab provided sustained, clinically meaningful improvements in fatigue. Our results contribute to the consistency and totality of the data demonstrating that eculizumab lowers the clinical burden in patients with AChR+ refractory gMG.

## Electronic supplementary material

Below is the link to the electronic supplementary material.

Supplementary material 1 (DOCX 34 KB)
