# Correction to: Eculizumab improves fatigue in refractory generalized myasthenia gravis

**DOI:** 10.1007/s11136-019-02204-x

**Published:** 2019-05-21

**Authors:** Henning Andersen, Renato Mantegazza, Jing Jing Wang, Fanny O’Brien, Kaushik Patra, James F. Howard, The REGAIN Study Group, Claudio Gabriel Mazia, Miguel Wilken, Fabio Barroso, Juliet Saba, Marcelo Rugiero, Mariela Bettini, Marcelo Chaves, Gonzalo Vidal, Alejandra Dalila Garcia, Jan De Bleecker, Guy Van den Abeele, Kathy de Koning, Katrien De Mey, Rudy Mercelis, Délphine Mahieu, Linda Wagemaekers, Philip Van Damme, Annelies Depreitere, Caroline Schotte, Charlotte Smetcoren, Olivier Stevens, Sien Van Daele, Nicolas Vandenbussche, Annelies Vanhee, Sarah Verjans, Jan Vynckier, Ann D’Hondt, Petra Tilkin, Alzira Alves de Siqueira Carvalho, Igor Dias Brockhausen, David Feder, Daniel Ambrosio, Pamela César, Ana Paula Melo, Renata Martins Ribeiro, Rosana Rocha, Bruno Bezerra Rosa, Thabata Veiga, Luiz Augusto da Silva, Murilo Santos Engel, Jordana Gonçalves Geraldo, Maria da Penha Ananias Morita, Erica Nogueira Coelho, Gabriel Paiva, Marina Pozo, Natalia Prando, Debora Dada Martineli Torres, Cristiani Fernanda Butinhao, Gustavo Duran, Tamires Cristina Gomes da Silva, Luiz Otavio Maia Gonçalves, Lucas Eduardo Pazetto, Tomás Augusto Suriane Fialho, Luciana Renata Cubas Volpe, Luciana Souza Duca, Maurício André Gheller Friedrich, Alexandre Guerreiro, Henrique Mohr, Maurer Pereira Martins, Daiane da Cruz Pacheco, Luciana Ferreira, Ana Paula Macagnan, Graziela Pinto, Aline de Cassia Santos, Acary Souza Bulle Oliveira, Ana Carolina Amaral Andrade, Marcelo Annes, Liene Duarte Silva, Valeria Cavalcante Lino, Wladimir Pinto, Natália Assis, Fernanda Carrara, Carolina Miranda, Iandra Souza, Patricia Fernandes, Zaeem Siddiqi, Cecile Phan, Jeffrey Narayan, Derrick Blackmore, Ashley Mallon, Rikki Roderus, Elizabeth Watt, Stanislav Vohanka, Josef Bednarik, Magda Chmelikova, Marek Cierny, Stanislava Toncrova, Jana Junkerova, Barbora Kurkova, Katarina Reguliova, Olga Zapletalova, Jiri Pitha, Iveta Novakova, Michaela Tyblova, Ivana Jurajdova, Marcela Wolfova, Thomas Harbo, Lotte Vinge, Susanne Krogh, Anita Mogensen, John Vissing, Joan Højgaard, Nanna Witting, Anne Ostergaard Autzen, Jane Pedersen, Juha-Pekka Eralinna, Mikko Laaksonen, Olli Oksaranta, Tuula Harrison, Jaana Eriksson, Csilla Rozsa, Melinda Horvath, Gabor Lovas, Judit Matolcsi, Gyorgyi Szabo, Gedeonne Jakab, Brigitta Szabadosne, Laszlo Vecsei, Livia Dezsi, Edina Varga, Monika Konyane, Giovanni Antonini, Antonella Di Pasquale, Matteo Garibaldi, Stefania Morino, Fernanda Troili, Laura Fionda, Allessandro Filla, Teresa Costabile, Enrico Marano, Francesco Saccà, Angiola Fasanaro, Angela Marsili, Giorgia Puorro, Carlo Antozzi, Silvia Bonanno, Giorgia Camera, Alberta Locatelli, Lorenzo Maggi, Maria Pasanisi, Angela Campanella, Amelia Evoli, Paolo Emilio Alboini, Valentina D’Amato, Raffaele Iorio, Maurizio Inghilleri, Laura Fionda, Vittorio Frasca, Elena Giacomelli, Maria Gori, Diego Lopergolo, Emanuela Onesti, Vittorio Frasca, Maria Gabriele, Akiyuki Uzawa, Tetsuya Kanai, Naoki Kawaguchi, Masahiro Mori, Yoko Kaneko, Akiko Kanzaki, Eri Kobayashi, Hiroyuki Murai, Katsuhisa Masaki, Dai Matsuse, Takuya Matsushita, Taira Uehara, Misa Shimpo, Maki Jingu, Keiko Kikutake, Yumiko Nakamura, Yoshiko Sano, Kimiaki Utsugisawa, Yuriko Nagane, Ikuko Kamegamori, Tomoko Tsuda, Yuko Fujii, Kazumi Futono, Yukiko Ozawa, Aya Mizugami, Yuka Saito, Hidekazu Suzuki, Miyuki Morikawa, Makoto Samukawa, Sachiko Kamakura, Eriko Miyawaki, Hirokazu Shiraishi, Teiichiro Mitazaki, Masakatsu Motomura, Akihiro Mukaino, Shunsuke Yoshimura, Shizuka Asada, Seiko Yoshida, Shoko Amamoto, Tomomi Kobashikawa, Megumi Koga, Yasuko Maeda, Kazumi Takada, Mihoko Takada, Masako Tsurumaru, Yumi Yamashita, Yasushi Suzuki, Tetsuya Akiyama, Koichi Narikawa, Ohito Tano, Kenichi Tsukita, Rikako Kurihara, Fumie Meguro, Yusuke Fukuda, Miwako Sato, Meinoshin Okumura, Soichiro Funaka, Tomohiro Kawamura, Masayuki Makamori, Masanori Takahashi, Namie Taichi, Tomoya Hasuike, Eriko Higuchi, Hisako Kobayashi, Kaori Osakada, Tomihiro Imai, Emiko Tsuda, Shun Shimohama, Takashi Hayashi, Shin Hisahara, Jun Kawamata, Takashi Murahara, Masaki Saitoh, Shuichiro Suzuki, Daisuke Yamamoto, Yoko Ishiyama, Naoko Ishiyama, Mayuko Noshiro, Rumi Takeyama, Kaori Uwasa, Ikuko Yasuda, Anneke van der Kooi, Marianne de Visser, Tamar Gibson, Byung-Jo Kim, Chang Nyoung Lee, Yong Seo Koo, Hung Youl Seok, Hoo Nam Kang, HyeJin Ra, Byoung Joon Kim, Eun Bin Cho, MiSong Choi, HyeLim Lee, Ju-Hong Min, Jinmyoung Seok, JiEun Lee, Da Yoon Koh, JuYoung Kwon, SangAe Park, Eun Hwa Choi, Yoon-Ho Hong, So-Hyun Ahn, Dae Lim Koo, Jae-Sung Lim, ChaeWon Shin, Ji Ye Hwang, Miri Kim, Seung Min Kim, Ha-Neul Jeong, JinWoo Jung, Yool-hee Kim, Hyung Seok Lee, Ha Young Shin, Eun Bi Hwang, Miju Shin, Carlos Casasnovas, Maria Antonia Alberti Aguilo, Christian Homedes-Pedret, Natalia Julia Palacios, Laura Diez Porras, Valentina Velez Santamaria, Ana Lazaro, Exuperio Diez Tejedor, Pilar Gomez Salcedo, Mireya Fernandez-Fournier, Pedro Lopez Ruiz, Francisco Javier Rodriguez de Rivera, Maria Sastre, Josep Gamez, Pilar Sune, Maria Salvado, Gisela Gili, Gonzalo Mazuela, Isabel Illa, Elena Cortes Vicente, Jordi Diaz-Manera, Luis Antonio Querol Gutierrez, Ricardo Rojas Garcia, Nuria Vidal, Elisabet Arribas-Ibar, Fredrik Piehl, Albert Hietala, Lena Bjarbo, Ihsan Sengun, Arzu Meherremova, Pinar Ozcelik, Bengu Balkan, Celal Tuga, Muzeyyen Ugur, Sevim Erdem-Ozdamar, Can Ebru Bekircan-Kurt, Nazire Pinar Acar, Ezgi Yilmaz, Yagmur Caliskan, Gulsah Orsel, Husnu Efendi, Seda Aydinlik, Hakan Cavus, Ayse Kutlu, Gulsar Becerikli, Cansu Semiz, Ozlem Tun, Murat Terzi, Baki Dogan, Musa Kazim Onar, Sedat Sen, Tugce Kirbas Cavdar, Adife Veske, Fiona Norwood, Aikaterini Dimitriou, Jakit Gollogly, Mohamed Mahdi-Rogers, Arshira Seddigh, Giannis Sokratous, Gal Maier, Faisal Sohail, Saiju Jacob, Girija Sadalage, Pravin Torane, Claire Brown, Amna Shah, Sivakumar Sathasivam, Heike Arndt, Debbie Davies, Dave Watling, Anthony Amato, Thomas Cochrane, Mohammed Salajegheh, Kristen Roe, Katherine Amato, Shirli Toska, Gil Wolfe, Nicholas Silvestri, Kara Patrick, Karen Zakalik, Jonathan Katz, Robert Miller, Marguerite Engel, Dallas Forshew, Elena Bravver, Benjamin Brooks, Sarah Plevka, Maryanne Burdette, Scott Cunningham, Mohammad Sanjak, Megan Kramer, Joanne Nemeth, Clara Schommer, Scott Tierney, Vern Juel, Jeffrey Guptill, Lisa Hobson-Webb, Janice Massey, Kate Beck, Donna Carnes, John Loor, Amanda Anderson, Robert Pascuzzi, Cynthia Bodkin, John Kincaid, Riley Snook, Sandra Guingrich, Angela Micheels, Vinay Chaudhry, Andrea Corse, Betsy Mosmiller, Andrea Kelley, Doreen Ho, Jayashri Srinivasan, Michal Vytopil, Jordan Jara, Nicholas Ventura, Stephanie Scala, Cynthia Carter, Craig Donahue, Carol Herbert, Elaine Weiner, Sharmeen Alam, Jonathan McKinnon, Laura Haar, Naya McKinnon, Karan Alcon, Kaitlyn McKenna, Nadia Sattar, Kevin Daniels, Dennis Jeffery, John Kissel, Miriam Freimer, Joseph Chad Hoyle, Julie Agriesti, Sharon Chelnick, Louisa Mezache, Colleen Pineda, Filiz Muharrem, Chafic Karam, Julie Khoury, Tessa Marburger, Harpreet Kaur, Diana Dimitrova, James Gilchrist, Brajesh Agrawal, Mona Elsayed, Stephanie Kohlrus, Angela Andoin, Taylor Darnell, Laura Golden, Barbara Lokaitis, Jenna Seelback, Srikanth Muppidi, Neelam Goyal, Sarada Sakamuri, Yuen T. So, Shirley Paulose, Sabrina Pol, Lesly Welsh, Ratna Bhavaraju-Sanka, Alejandro Tobon Gonzales, Lorraine Dishman, Floyd Jones, Anna Gonzalez, Patricia Padilla, Amy Saklad, Marcela Silva, Sharon Nations, Jaya Trivedi, Steve Hopkins, Mohamed Kazamel, Mohammad Alsharabati, Liang Lu, Kenkichi Nozaki, Sandi Mumfrey-Thomas, Amy Woodall, Tahseen Mozaffar, Tiyonnoh Cash, Namita Goyal, Gulmohor Roy, Veena Mathew, Fatima Maqsood, Brian Minton, H. James Jones, Jeffrey Rosenfeld, Rebekah Garcia, Laura Echevarria, Sonia Garcia, Michael Pulley, Shachie Aranke, Alan Ross Berger, Jaimin Shah, Yasmeen Shabbir, Lisa Smith, Mary Varghese, Laurie Gutmann, Ludwig Gutmann, Nivedita Jerath, Christopher Nance, Andrea Swenson, Heena Olalde, Nicole Kressin, Jeri Sieren, Richard Barohn, Mazen Dimachkie, Melanie Glenn, April McVey, Mamatha Pasnoor, Jeffery Statland, Junxia Wang, Tina Liu, Kelley Emmons, Nicole Jenci, Jerry Locheke, Alex Fondaw, Kathryn Johns, Gabrielle Rico, Maureen Walsh, Laura Herbelin, Charlene Hafer-Macko, Justin Kwan, Lindsay Zilliox, Karen Callison, Valerie Young, Beth DiSanzo, Kerry Naunton, Michael Benatar, Martin Bilsker, Khema Sharma, Anne Cooley, Eliana Reyes, Sara-Claude Michon, Danielle Sheldon, Julie Steele, Chafic Karam, Manisha Chopra, Rebecca Traub, Tuan Vu, Lara Katzin, Terry McClain, Brittany Harvey, Adam Hart, Kristin Huynh, Said Beydoun, Amaiak Chilingaryan, Victor Doan, Brian Droker, Hui Gong, Sanaz Karimi, Frank Lin, Terry McClain, Krishna Pokala, Akshay Shah, Anh Tran, Salma Akhter, Ali Malekniazi, Rup Tandan, Michael Hehir, Waqar Waheed, Shannon Lucy, Michael Weiss, Jane Distad, Susan Strom, Sharon Downing, Bryan Kim, Tulio Bertorini, Thomas Arnold, Kendrick Hendersen, Rekha Pillai, Ye Liu, Lauren Wheeler, Jasmine Hewlett, Mollie Vanderhook, Richard Nowak, Daniel Dicapua, Benison Keung, Aditya Kumar, Huned Patwa, Kimberly Robeson, Irene Yang, Joan Nye, Hong Vu

**Affiliations:** 1 Department of Neurology, Aarhus University Hospital, Indgang J, Plan 5, Krydspunkt 504, Palle Juul-Jensens Boulevard, 8200 Aarhus N, Denmark; 2 Foundation of the Carlo Besta Neurological Institute, IRCCS, Milan, Italy; 3 Formerly of Alexion Pharmaceuticals, Inc., New Haven, CT USA; 4 Alexion Pharmaceuticals, Inc., New Haven, CT USA; 5 Department of Neurology, UNC School of Medicine, University of North Carolina at Chapel Hill, Chapel Hill, NC USA

## Correction to: Quality of Life Research 10.1007/s11136-019-02148-2

The article “Eculizumab improves fatigue in refractory generalized myasthenia gravis”, written by “Henning Andersen, Renato Mantegazza, Jing Jing Wang, Fanny O’Brien, Kaushik Patra, James F. Howard Jr. and The REGAIN Study Group” was originally published electronically on the publisher’s internet portal (currently SpringerLink) on 23 March 2019 without open access.

With the author(s)’ decision to opt for Open Choice the copyright of the article changed on 20 May 2019 to © The Author(s) 2019 and the article is forthwith distributed under the terms of the Creative Commons Attribution 4.0 International License (http://creativecommons.org/licenses/by/4.0/), which permits use, duplication, adaptation, distribution and reproduction in any medium or format, as long as you give appropriate credit to the original author(s) and the source, provide a link to the Creative Commons license and indicate if changes were made.

The original article has been corrected.

